# Machine Learning Prediction of Treatment Response to Biological Disease-Modifying Antirheumatic Drugs in Rheumatoid Arthritis

**DOI:** 10.3390/jcm13133890

**Published:** 2024-07-02

**Authors:** Fatemeh Salehi, Luis I. Lopera Gonzalez, Sara Bayat, Arnd Kleyer, Dario Zanca, Alexander Brost, Georg Schett, Bjoern M. Eskofier

**Affiliations:** 1 Machine Learning and Data Analytics Laboratory, Department Artificial Intelligence in Biomedical Engineering, Friedrich Alexander University Erlangen-Nuremberg, 91052 Erlangen, Germany; dario.zanca@fau.de (D.Z.); bjoern.eskofier@fau.de (B.M.E.); 2 Instutue of Digital Health, Friedrich Alexander University Erlangen-Nuremberg, 91052 Erlangen, Germany; luis.i.lopera@fau.de; 3 Department of Internal Medicine 3, Rheumatology and Immunology, University Hospital Erlangen, 91054 Erlangen, Germany; sara.bayat@uk-erlangen.de (S.B.); georg.schett@uk-erlangen.de (G.S.); 4 Deutsches Zentrum Immuntherapie (DZI), 91054 Erlangen, Germany; 5 Department of Rheumatology and Clinical Immunology, Charité—University Medicine Berlin, 10117 Berlin, Germany; arnd.kleyer@extern.uk-erlangen.de; 6 Siemens Healthcare GmbH, 91301 Forchheim, Germany; alexander.brost@siemens-healthineers.com; 7 Translational Digital Health Group, Institute of AI for Health, Helmholtz Center Munich—German Research Center for Environmental Health, 85764 Neuherberg, Germany

**Keywords:** bDMARDs, machine learning predictive model, rheumatoid arthritis, treatment response, prediction

## Abstract

**Background:** Disease-modifying antirheumatic drugs (bDMARDs) have shown efficacy in treating Rheumatoid Arthritis (RA). Predicting treatment outcomes for RA is crucial as approximately 30% of patients do not respond to bDMARDs and only half achieve a sustained response. This study aims to leverage machine learning to predict both initial response at 6 months and sustained response at 12 months using baseline clinical data. **Methods:** Baseline clinical data were collected from 154 RA patients treated at the University Hospital in Erlangen, Germany. Five machine learning models were compared: Extreme Gradient Boosting (XGBoost), Adaptive Boosting (AdaBoost), K-nearest neighbors (KNN), Support Vector Machines (SVM), and Random Forest. Nested cross-validation was employed to ensure robustness and avoid overfitting, integrating hyperparameter tuning within its process. **Results:** XGBoost achieved the highest accuracy for predicting initial response (AUC-ROC of 0.91), while AdaBoost was the most effective for sustained response (AUC-ROC of 0.84). Key predictors included the Disease Activity Score-28 using erythrocyte sedimentation rate (DAS28-ESR), with higher scores at baseline associated with lower response chances at 6 and 12 months. Shapley additive explanations (SHAP) identified the most important baseline features and visualized their directional effects on treatment response and sustained response. **Conclusions:** These findings can enhance RA treatment plans and support clinical decision-making, ultimately improving patient outcomes by predicting response before starting medication.

## 1. Introduction

Rheumatoid Arthritis (RA) is a common inflammatory condition that primarily affects the small joints of the hands and feet, leading to disability, discomfort and deformity. It affects approximately 0.5–1% of the global population [1,2,3].

Biological disease-modifying antirheumatic drugs (bDMARDs) are an effective treatment for RA, typically prescribed when patients do not adequately respond to conventional synthetic DMARDs (csDMARDs) [4]. According to the European Alliance of Associations for Rheumatology (EULAR) recommendations, regular monitoring every 1–3 months is essential in managing active RA. Therapy should be adjusted if a response is not achieved within six months [5]. The goal of RA treatment is a sustained response, defined as remaining in remission or at least low disease activity (LDA). Patients on bDMARDs should have at least two follow-up visits within six months after achieving a response to ensure sustained response [6]. Despite their effectiveness, 30–40% of patients do not respond to bDMARDs, and only 50% achieve a sustained response [7,8]. Early response prediction before starting medication and appropriate treatment selection can improve disease control, reduce joint damage and alleviate pain [9,10]. Non-responders may experience uncontrolled disease progression, leading to increased healthcare costs and a decline in quality of life [11,12,13,14]. Although the costs of bDMARDs have decreased, their substantial cost still necessitates judicious use of healthcare resources [15].

Machine learning (ML) can identify patterns and relationships within data, offering potential benefits for predicting bDMARD outcomes and helping rheumatologists make more accurate treatment decisions [16]. While previous studies have explored ML techniques for predicting treatment response in RA patients, they face several challenges, such as limitations associated with the availability and cost of imaging and gene expression data [17]. For example, San Koo et al. [18] and Lee et al. [19] used clinical and imaging data to predict response at one year, but imaging data are often not available in routine practice and one year is too long to wait for therapy adjustment. Guan et al. [20] used clinical and genetic markers to predict response over 24 months, but genetic data are typically not available in routine clinical practice. Tao et al. [21] focused on predicting response at six months using genetic data, which is also often inaccessible. In addition, Rivellese et al. [22] and Yoosuf et al. [23] used gene expression to predict treatment response. However, such datasets are not available for every patient and are expensive for healthcare systems. To our knowledge, none of this research has predicted the sustained response at twelve months.

This study aims to overcome existing limitations by using routine clinical data before starting medication, to predict both the initial response and the sustained response. Moreover, we identify the most relevant baseline clinical features and their directional effects on treatment outcomes using Shapley additive explanations (SHAP), which enhances the model’s interpretability for physicians. Our approach not only aligns with the EULAR gold standard for six-month response but also focuses on the sustained response, a critical yet often neglected aspect of RA treatment. We assess our strategy using clinical data from RA patients treated with bDMARDs, incorporating baseline and follow-up information from the initiation of treatment until any change or discontinuation of therapy.

## 2. Materials and Methods

### 2.1. Data Collection

In this study, we collected anonymized data from RA patients at Erlangen University Hospital in Germany. All patients met the ACR/EULAR 2010 classification criteria for RA [24]. The research conducted in this study complied entirely with the principles outlined in the Declaration of Helsinki. The ethics committee of Friedrich-Alexander University (FAU) approved conducting the research in a cohort of patients with RA, with approval reference numbers 334-18 B and 333-16 B.

These patients were included from the time they initiated the bDMARDs treatment until they either changed the treatment or tapered these medications. For each patient, the study gathered clinical data for a baseline established at the time the patient started taking bDMARDs. Subsequent data were collected during patient follow-ups. All gathered clinical characteristics followed the same healthcare protocols and guidelines throughout the entirety of the study period. Demographic characteristics like age and gender, as well as disease-specific characteristics such as the type of medications the patients were taking in addition to their bDMARDs, like csDMARDs and non-steroidal anti-inflammatory drugs (NSAIDs), were recorded. Disease-specific characteristics also included C-reactive protein (CRP) level, erythrocyte sedimentation rate (ESR), rheumatoid factor (RF) and presence of cyclic citrullinated peptide (CCP) antibodies. Furthermore, disease activity measures, such as tender and swollen joint counts based on 28 joints (TJC28 and SJC28), visual analogue scales (VAS) for pain, patients and physicians’ global disease activity, disease activity score in 28 joints based on CRP and ESR (DAS28-ESR and DAS28-CRP), clinical disease activity index (CDAI), simple disease activity Index (SDAI) and health assessment questionnaire (HAQ) were assessed. The study also took into account other comorbidities, for instance, asthma, diabetes, heart disease, etc. The list of all features can be seen in Table A2 and Table A3.

### 2.2. Data Preprocessing

Before analyzing the collected data, a preprocessing step was necessary to address inconsistencies due to the routine data-collection process. It is important to note that rheumatologists collected data during their own diagnoses and treatments. However, some challenges emerged, such as the selective collection of features during follow-up visits. In some cases, specific features were recorded only once (rather than during every follow-up), like, for example, gender or certain features might have been omitted intentionally or accidentally, leading to various missing values in the raw data. To overcome this issue, we implemented a comprehensive imputation strategy. The proportion of missing data for each value is summarized in Table A1.

For the imputation of missing values, we employed different methods depending on the variable characteristics. For variables demonstrating linear correlations with other follow-up measurements, such as patient comorbidities, we utilized straightforward linear imputation techniques like the nearest available observation (NAO) and linear extrapolation [25]. These imputations were conducted on an individual patient basis and remained independent of data from other patients.

For other missing values, such as DAS28 and the values used to calculate DAS28 (e.g., CDI Score, SDI Score SJC28 and TJC28), which did not show linear correlations with other variables, we used the Multiple Imputation by Chained Equations (MICE) method [26]. MICE uses data from various variables to estimate the best possible prediction for each missing value by considering data from all patients collectively, not just individually.

We chose MICE because it can also handle datasets with up to 20% missing data. It creates multiple imputations for missing values by modeling each variable with missing data based on other variables in the dataset [27,28]. This method ensures that the imputed values are as accurate as possible, maintaining the dataset’s integrity for analysis [29].

Using imputation was crucial to avoid biases and inaccuracies from incomplete data, ensuring our analyses were robust and reliable.

### 2.3. Response and Sustained Response Groups

In this study, we defined responses to bDMARDs into two states: remission and low disease activity (LDA) [30]. The sustained response also refers to the maintenance of remission or LDA states for at least six months [6]. Based on the EULAR criteria, we used DAS28 score based on the erythrocyte sedimentation rate (DAS28-ESR) score to quantify remission and LDA. A DAS28-ESR score below 2.6 indicates remission, while a score between 2.6 and 3.2 indicates LDA [31].

In evaluating the effectiveness of bDMARDs treatment, patients were categorized based on their response and sustained response to the treatment. To assess the response, patients were labeled as “responders” if they met the remission or LDA criteria after 6 months and if they could maintain these criteria for an additional six months, requiring at least two visits within this period to be considered part of the sustained responder group. Patients who did not meet the responder criteria were categorized as “non-sustained responders”, regardless of their DAS28-ESR values within the second six months.

### 2.4. Predictive Classification Models

**Figure 1:**
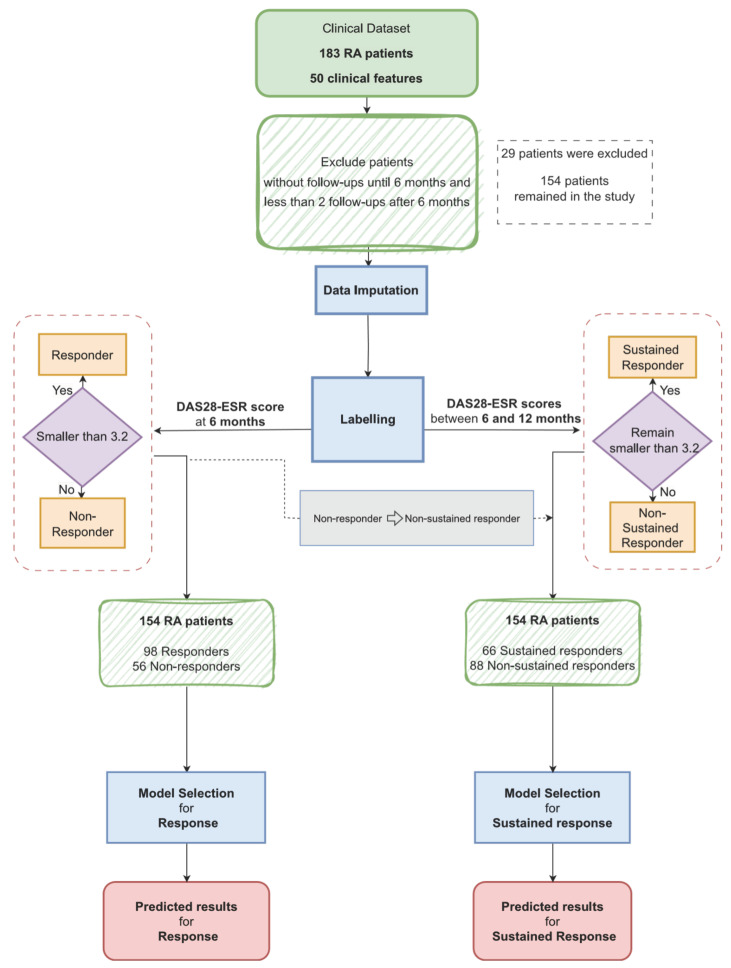
Data-processing flowchart showing patient selection, labeling strategy and delivery to the respective prediction model-selection units.

In order to predict patient response and sustained response to bDMARDs treatment, we employed two separate machine learning models, as illustrated in Figure 1. Both models utilize the same clinical data to predict the patient’s response to treatment at the six-month follow-up and estimate their sustained response to treatment after twelve months, respectively. For both models, we provide information regarding the most important clinical features influencing the model’s outcome. We evaluated multiple classification models to identify the most effective approach. Five machine learning classifiers were trained with the selected features: Support Vector Machine (SVM) [32], Random Forest [33], Extreme Gradient Boosting (XGBoost) [34], Adaptive Boosting (AdaBoost) [35] and K-nearest neighbor (KNN) [36].

### 2.5. Feature Importance and Interpretability

To make our models more reliable and easier to understand, we found and selected the most important features. We used a technique called Random Forest (RF) to figure out which features were crucial [37]. Then, we ranked them based on their importance. Nevertheless, the RF algorithm alone does not provide information regarding the direction in which these variables influence the outcome of predictions. To address the problem, we used Shapley additive explanations (SHAP) [38,39]. SHAP computed the difference in model output with and without each feature. This resulted in a SHapley value for each feature, which not only indicated its contribution but also the direction of its effect on the model’s predictions. This information is crucial for clinicians to identify the best variables that significantly influence the prediction of response and sustained response in bDMARDs treatment.

### 2.6. Model Selection

We used a nested cross-validation methodology for training, validating and testing prediction models [40]. This technique guarantees our models’ robustness and best performance when processing real-world clinical data, which is essential for healthcare applications.

The nested cross-validation process consists of two main parts: an outer loop and an inner loop. In the framework of the outer loop, we first split our dataset into two main segments: an 80% training set and a 20% test set.

The training data were subsequently split into five different subsets, or “folds”, within the inner loop. Here, we used hyperparameter tuning and feature selection. Using four folds for training and one for validation, we explored different combinations of features and hyperparameters in each iteration. After choosing the features, we used a grid search for each classifier, assessing every possible combination of hyperparameters. The search for the selection of the best hyperparameters was based on achieving the maximum accuracy on the validation dataset. This procedure was iterated five times, ultimately identifying the most effective hyperparameters and features for classifiers. Table A4 provides a list of the hyperparameters and the search space for each classifier.

In the second stage, we trained and evaluated our classifiers within the outer loop of the nested cross-validation method, utilizing the chosen features and the best hyperparameters identified in the first step. In this phase, we assessed performance metrics using the test dataset.

**Figure 2:**
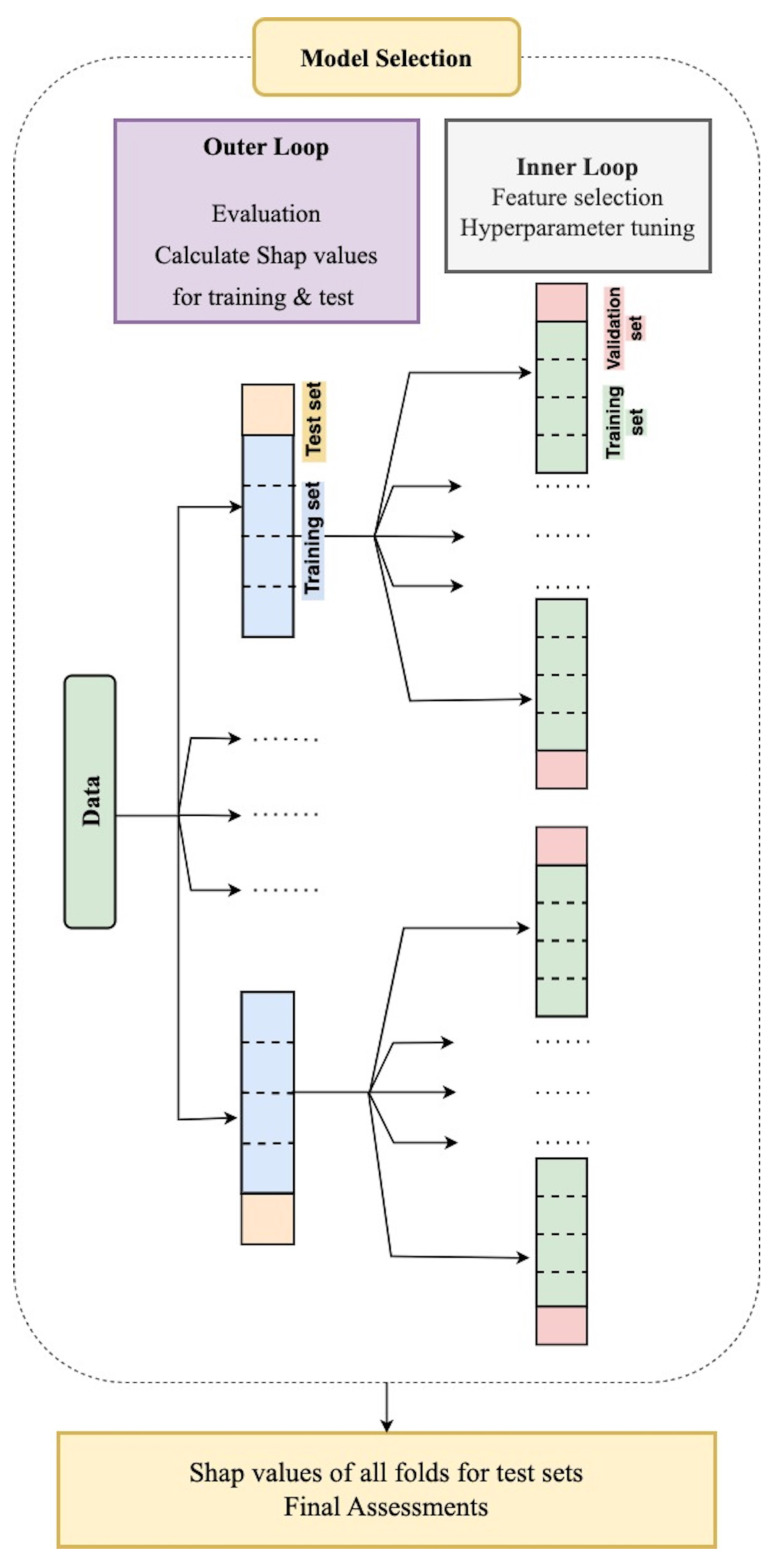
Flowchart illustrating the nested cross-validation process for feature selection, hyperparameter tuning and model selection. The nested cross-validation consists of five outer loops and five inner loops. In the inner loop, the optimal combination of features and hyperparameters is determined. During each round of the outer cross-validation, the SHAP values of the selected features are calculated on the test folds (Image adapted from [42]).

We repeated this method four times, using one of the remaining test folds as the testing dataset and the other as the training dataset in each iteration. For each new training dataset, the inner loop is repeated five times, resulting in a 5 × 5 cross-validation process. In addition, within the outer loop, we computed SHAP values for the features that were chosen [41]. After concatenating these SHAP values, we were able to identify the most significant features in our models. Figure 2 presents a visual representation of this nested cross-validation process.

To evaluate the performance of the classifiers in predicting response and sustained response, we utilized four evaluation metrics: accuracy, Area Under the Receiver Operating Characteristic Curve (AUC-ROC), Matthews Correlation Coefficient (MCC) and F1 score. The cut-off threshold for the ROC curves was set to 0.5. This threshold is commonly chosen as it assumes equal costs for false positives and false negatives.

Using the outer loop of nested cross-validation, we obtained five values of each metric for each classifier prediction. The best classifiers for response and sustained response prediction were selected based on a combination of the highest mean of the evaluation metrics and also taking into account standard deviation as an indicator of variability. This approach allowed us to prioritize classifiers with both strong average performance and relatively low variability.

### 2.7. Software

The machine learning models and analysis scripts used in this study were developed using Python 3.9. The code and libraries used in this study are available on GitHub.

## 3. Results

### 3.1. Patients Characteristics

Among the 183 RA patients screened, 154 had at least one follow-up at six months from baseline and at least two follow-ups within the subsequent six months. The remaining 29 patients were excluded as they did not meet these criteria. Table A2 presents the baseline clinical features of these 154 RA patients, stratified by their response at six months. Additionally, Table A3 provides a stratification based on sustained response.

**Figure 3:**
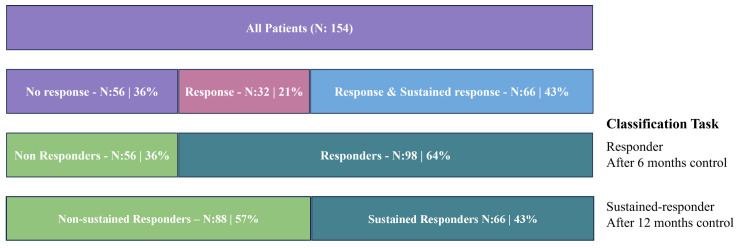
The proportion of responders and non-responders (after 6 months), and sustained responders and non-sustained responders (after 12 months).

Out of 154 RA patients, 98 were identified as responders to the treatment at the six-month follow-up, and subsequently, 66 of these responders were recognized as having sustained response. The distribution of patients across these groups is illustrated in Figure 3, highlighting that while 64% of patients met the response criteria, only 43% were able to maintain a sustained response.

The patients were supplied various bDMARDs, including Etanercept, Adalimumab, Certolizumab, Rituximab, Infliximab, Abatacept, Tocilizumab, Golimumab, Sarilumab, Secukinumab, Anakinra and Ustekinumab.

#### 3.1.1. bDMARDs Response Prediction

We assessed five classifiers to predict patient response to bDMARDs treatment and classified them into two groups: responder and non-responder. Our dataset of 154 RA patients was divided into folds within the inner loop of the nested cross-validation process, with roughly 31 patients included for training and validation for each fold. This distribution meant that, for the outer loop, roughly 123 patients (80% of the total of 154), divided into five folds for the inner loop, were utilized for training in each of the five iterations, while the remaining 31 patients (20% of the total of 154), formed the test set. This made sure that every patient was fairly represented during the training and testing phases enabling thorough evaluation of the model’s functionality.

**Figure A1:**
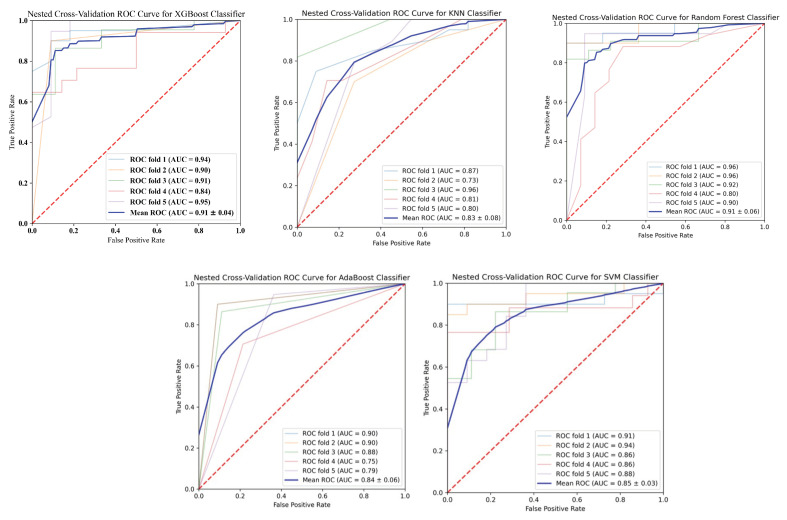
ROC Curves for Nested Cross-Validation: Each curve represents the ROC for a test fold in the outer loop of the nested cross-validation process used for predicting response at six months.

From all classifiers, XGBoost outperformed the others, achieving the highest accuracy, AUC-ROC, MCC and F1 score. XGBoost demonstrated mean values of 0.851, 0.91, 0.714 and 0.878 for accuracy, AUC-ROC, MCC and F1 score, respectively (Table 1). Figure A1 shows the ROC curves for the five outer test folds of the classifiers.

**Figure 4:**
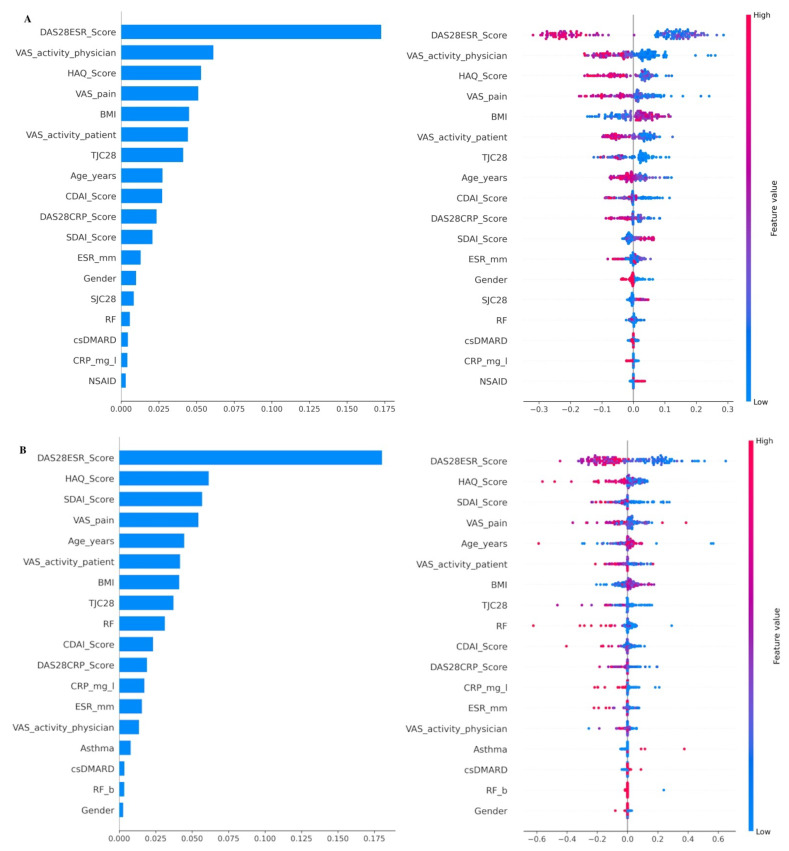
The figure shows the most effective baseline features and their impact in predicting responses (**A**) using the XGBoost classifier, as well as the sustained response (**B**) through the implementation of AdaBoost classifiers. The SHAP values for the chosen features, which were obtained by the Random Forest (RF) model, are shown for predicting both the response and the sustained response. The figures on the left side show the average of absolute SHAP values and the mean influence of these features on the model. In contrast, the figures on the right side illustrate SHAP values, where positive values indicate a higher likelihood of response and sustained response, while negative values suggest the opposite. Vice versa, smaller SHAP values correspond to a lower probability of response and sustained response. The model for each patient is visually represented by a dot, where dots with greater SHAP values are shown in red and dots with lower values are represented in blue. For binary variables such as gender and diseases (e.g., male/female and presence/absence of disease, respectively), red dots indicate the presence of the condition or female gender, while blue dots represent the absence of the condition or male gender. The y-axis shows the most significant clinical features at the baseline for making these predictions.

Additionally, Figure 4 displays the SHAP plots. The figure on the left side of Figure 4A displays the mean of absolute SHAP values, which shows the baseline features in descending order of importance for predicting the response to treatment using XGBoost. The key clinical features that are crucial for predicting treatment response after six months include DAS28-ESR, VAS for physician, HAQ score, VAS for pain assessment, Body Mass Index (BMI), VAS for patient, TJC28, age, CDAI score, SDAI score, ESR level, gender, SJC28, RF, csDMARDs taking, CRP level and NSAID usage, respectively.

The right plot visually represents the direction of each mentioned baseline feature using a dot distribution. Red dots indicate greater values, while blue dots indicate lower values. Each dot represents an individual patient. Positive SHAP values show a response, whereas negative SHAP values indicate no response. For example, let us consider the DAS28-ESR score, which is recognized as the most important feature for determining response. There is a positive correlation between lower values of DAS28-ESR and a higher probability of being classified as a responder after 6 months of therapy with bDMARDs.

#### 3.1.2. bDMARDs Sustained Response Prediction

**Figure A2:**
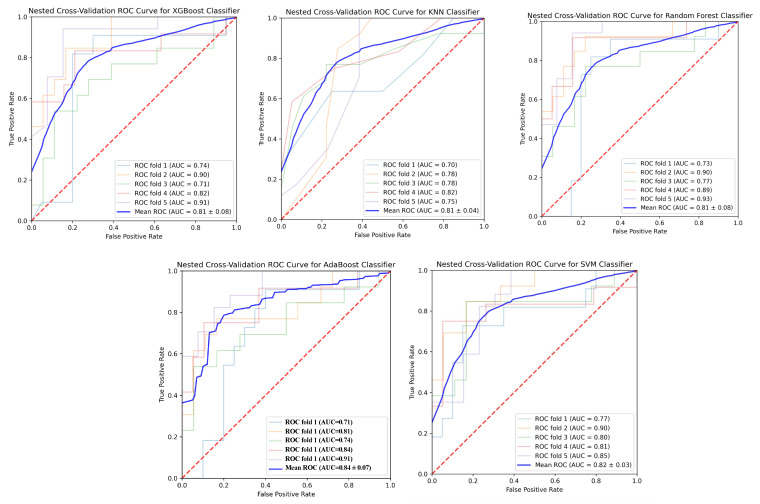
ROC Curves for Nested Cross-Validation: Each curve represents the ROC for a test fold in the outer loop of the nested cross-validation process for predicting sustained response at 12 months.

We also used five classifiers to predict sustained response to bDMARDs treatment. The assessment metrics presented in Table 2 reveal that the AdaBoost classifier achieved the highest mean accuracy, AUC-ROC, MCC and F1-Score for distinguishing patients based on their sustained response. The mean values for AdaBoost were 0.856, 0.842, 0.68 and 0.759 for accuracy, AUC-ROC, MCC and F1-Score, respectively. Figure A2 shows the ROC curves for the classifiers across the five outer folds of the nested cross-validation.

Figure 4B presents the SHAP plots for the AdaBoost classifier, highlighting the significant baseline features and their impact on sustained responses. The primary features for predicting sustained response are listed in descending order of importance, including the DAS28-ESR Score, HAQ Score, SDAI Score, VAS pain, age, VAS for patients, BMI, TJC28, RF, CDAI Score, DAS28-CRP score, CRP level, ESR level, VAS for physicians, asthma status, csDMARD usage, RF and gender. The last four attributes have a negligible impact. As previously stated, the SHAP method enhances our comprehension of how features influence outcomes and provides a clearer indication of the most influential features by analyzing the distribution of red and blue dots. For example, patients with lower DAS28-ESR and HAQ scores at the start of treatment have a higher likelihood of achieving sustained response.

## 4. Discussion

Our study utilized routine clinical data and machine learning methods to predict the response to bDMARDs treatment at six months and the sustained treatment response after twelve months. We achieved high predictive accuracy, with the XGBoost classifier showing an AUC-ROC of 0.910 for initial response prediction and the AdaBoost classifier demonstrating an AUC-ROC of 0.842 for sustained response. The robustness of our models was ensured through nested cross-validation, with SHAP values providing insights into feature importance and directionality.

Using baseline data, we can predict patient outcomes before starting the medication, allowing for more accurate and efficient care by quickly identifying patients unlikely to benefit from these therapies. This approach optimizes follow-up schedules, improves overall treatment effectiveness and better allocates healthcare resources.

Our study addresses gaps in previous research by aligning endpoints with EULAR standards and focusing on sustained response, thus enhancing the practicality and applicability of our findings. The emphasis on baseline characteristics provides clinicians with valuable guidance for making informed treatment decisions before prescribing medication. This targeted approach is crucial for effective patient management in rheumatology.

Despite these advances, our study has certain limitations. Its single-center nature may limit the generalizability of the findings. Future research should validate these predictive models in diverse settings and among larger patient groups to confirm their broader applicability and strengthen their predictive power. Additionally, expanding the scope of our models to include a wider range of DMARDs could significantly enhance the ability to tailor treatment plans. By extending the predictive capabilities to various DMARD categories, we aim to provide clinicians with a comprehensive decision-making tool, ensuring each patient receives the most suitable and effective treatment.

In conclusion, our study presents a promising approach for predicting bDMARDs response and sustained response using routine clinical data. By highlighting the most critical features and explaining how each clinical feature can influence response and sustained response, our predictive models can serve as decision support tools to help rheumatologists make more informed decisions when prescribing bDMARDs.

## Figures and Tables

**Table 1:** Results of predictive classifiers of response.

Classifier	Accuracy	AUC-ROC	F1 Score	MCC
AdaBoost	0.808 (0.070)	0.849 (0.060)	0.872 (0.063)	0.686 (0.114)
SVM	0.812 (0.046)	0.848 (0.034)	0.851 (0.048)	0.490 (0.121)
KNN	0.766 (0.059)	0.827 (0.081)	0.821 (0.043)	0.366 (0.135)
**XGBoost**	**0.851 (0.044)**	**0.910 (0.040)**	**0.878 (0.053)**	**0.714 (0.179)**
Random Forest	0.852 (0.033)	0.908 (0.065)	0.846 (0.071)	0.640 (0.103)

**Note:** Mean (±SD) evaluation scores of classifiers that classify the RA patients into two groups (Responder and Non-responder to bDMARDs). **XGBoost** is the best classifier for classifying responses.

**Table 2:** Results of predictive classifiers of sustained response.

Classifier	Accuracy	AUC-ROC	F1 Score	MCC
**AdaBoost**	**0.856 (0.045)**	**0.842 (0.073)**	**0.759 (0.047)**	**0.680 (0.142)**
SVM	0.773 (0.054)	0.828 (0.034)	0.755 (0.065)	0.395 (0.202)
KNN	0.701 (0.105)	0.813 (0.040)	0.660 (0.097)	0.203 (0.105)
XGBoost	0.748 (0.091)	0.817 (0.080)	0.689 (0.124)	0.489 (0.227)
Random Forest	0.780 (0.054)	0.810 (0.081)	0.719 (0.100)	0.542 (0.215)

**Note:** Mean (±SD) evaluation scores of classifiers that classify the RA patients into two groups (Responder and Non-responder to bDMARDs). **AdaBoost** is the best classifier for classifying responses.

## Data Availability

The datasets generated and analyzed during the current study are available in Zenodo at the following URL: https://doi.org/10.5281/zenodo.12507169 (accessed on 25 June 2024). These datasets include all clinical baseline data and labelled data based on response and sustained response. Data were uploaded to the repository during the peer review process.

## Abbreviations

The following abbreviations are used in this manuscript:

ACR	American College Testing
AdaBoost	Adaptive Boosting
AUC-ROC	Area under the Receiver Operating Characteristic Curve
bDMARDs	Biological Disease-Modifying Antirheumatic Drugs
BMI	Body Mass Index
CDAI	Clinical Disease Activity Index
CCP	Cyclic Citrullinated Peptide Antibodies
CRP	C-Reactive Protein
csDMARDs	Conventional Disease-Modifying Antirheumatic Drugs
DAS28-CRP	Disease Activity Score-28 using C-Reactive Protein
DAS28-ESR	Disease Activity Score-28 using Erythrocyte Sedimentation Rate
ESR	Erythrocyte Sedimentation Rate
EULAR	European Alliance of Associations for Rheumatology
HAQ	Health Assessment Questionnaire
KNN	K-nearest Neighbors
KOBIO	Korean College of Rheumatology Biologics and Targeted Therapy Registry
LDA	Low Disease Activity
ML	Machine Learning
MICE	Multiple Imputations by Chained Equation
NAO	Nearest Available Observation
NSAIDs	Non-steroidal Anti-inflammatory Drugs
RA	Rheumatoid Arthritis
RF	Rheumatoid Factor
RF (Model)	Random Forest
SD	Standard Deviation
SDAI	Simple Disease Activity Index
SHAP	Shapley Additive Explanations
SJC28	Swollen Joint Count based on 28 joints
SVM	Support Vector Machines
TJC28	Tender Joint Count based on 28 joints
VAS	Visual Analogue Scales
XGBoost	Extreme Gradient Boosting
MCC	Matthews Correlation Coefficient

## Appendix A

**Table A1:** Percentage of Missing Data for Each Clinical Feature at Baseline.

Column	Percentage Missing (%)
Age (years)	0.00
Gender	0.00
Swollen Joint Count-28 (SJC28)	1.76
Tender Joint Count-28 (TJC28)	1.76
Body Mass Index (BMI)	0.00
Visual Analog Scale (VAS) Activity (Physician)	14.88
Visual Analog Scale (VAS) Activity (Patient)	1.76
Visual Analog Scale (VAS) Pain	14.18
Health Assessment Questionnaire (HAQ) Score	1.77
Disease Activity Score in 28 Joints (DAS28-ESR)	1.76
Disease Activity Score in 28 Joints (DAS28-CRP)	2.26
C-Reactive Protein (CRP) (mg/L)	0.00
Rheumatoid Factor (RF)	0.00
Anti-Cyclic Citrullinated Peptide (CCP)	0.00
Clinical Disease Activity Index (CDAI)	14.88
Simple Disease Activity Index (SDAI)	16.18
Erythrocyte Sedimentation Rate (ESR) (mm)	1.76
Osteoarthritis	0.00
Asthma	0.00
Uveitis	0.00
Hypertension	0.00
Chronic Renal Insufficiency	0.00
COPD	0.00
Depression	0.00
Diabetes	0.00
Inflammatory Bowel Disease	0.00
Fat Metabolism Disorder	0.00
Gout	0.00
Heart Attack	0.00
Coronary Heart Disease	0.00
Osteoporosis	0.00
Periodontitis	0.00
Thyroid Disease	0.00
Thrombosis	0.00
bDMARD	0.00
tsDMARD	0.00
csDMARD	0.00
Prednisolone	0.00
Non-Steroidal Anti-Inflammatory Drug (NSAID)	0.00
bDMARD Intake Duration (days)	0.00

**Table A2:** Baseline Clinical Features of RA Patients, Stratified by Response to bDMARDs After 6 Months.

Baseline Characteristics	Responders (*n* = 98)	Non-Responders (*n* = 56)	*p*-Value
Age (years)	51.86 (13.95)	56.29 (11.75)	0.04696
Gender (Female)%	67.35%	82.14%	1.00000
Body Mass Index (BMI)	28.34 (17.26)	27.24 (5.94)	0.64452
Anti-Cyclic Citrullinated Peptide (CCP)	211.68 (313.09)	283.36 (528.09)	0.29154
Clinical Disease Activity Index (CDAI)	5.41 (6.47)	16.41 (11.89)	0.00000
C-Reactive Protein (CRP) (mg/L)	0.32 (0.47)	0.41 (0.5)	0.24045
Health Assessment Questionnaire (HAQ) Score	0.62 (0.66)	0.94 (0.68)	0.00000
Non-Steroidal Anti-Inflammatory Drug (NSAID) Usage%	27.55%	28.57%	1.00000
Rheumatoid Factor (RF)	71.72 (125.38)	145.58 (267.65)	0.02136
Simple Disease Activity Index (SDAI)	6.72 (7.37)	17.12 (11.63)	0.00000
Swollen Joint Count-28 (SJC28)	1.58 (3.47)	3.62 (4.85)	0.00283
Tender Joint Count-28 (TJC28)	1.43 (2.84)	6.79 (6.27)	0.00000
Visual Analog Scale (VAS) Activity (Patient)	20.68 (19.18)	53.0 (22.0)	0.00000
Visual Analog Scale (VAS) Activity (Physician)	11.52 (13.24)	32.95 (22.14)	0.00000
Visual Analog Scale (VAS) Pain	18.96 (18.04)	43.45 (23.11)	0.00000
Erythrocyte Sedimentation Rate (ESR) (mm)	12.71 (9.74)	25.12 (22.19)	0.00000
Disease Activity Score in 28 Joints (DAS28-CRP)	2.37 (0.86)	3.97 (1.13)	0.00000
Disease Activity Score in 28 Joints (DAS28-ESR)	2.39 (1.04)	4.38 (1.16)	0.00000
Asthma %	0.0%	0.0%	1.00000
Inflammatory Bowel Disease %	0.0%	0.0%	1.00000
Prednisolone%	28.57%	55.36%	1.00000
Chronic Renal Insufficiency %	0.0%	0.0%	1.00000
Coronary Heart Disease%	0.0%	0.0%	1.00000
Diabetes%	0.0%	0.0%	1.00000
Fat Metabolism Disorder%	0.0%	0.0%	1.00000
Gout%	0.0%	0.0%	1.00000
Conventional Synthetic Disease-Modifying Antirheumatic Drugs (csDMARD) %	65.31%	69.64%	1.00000

Mean (±standard deviation) and percentage of the population for each variable at baseline for RA patients treated with bDMARDs. ***p***-values indicate the statistical significance of differences between responders and non-responders.

**Table A3:** Baseline Clinical Features of RA Patients, Stratified by Sustained Response to bDMARDs After 12 Months.

	Sustained	Non-Sustained	
Baseline Characteristics	Responders (*n* = 66)	Responders (*n* = 88)	*p*-Value
Age (years)	51.52 (14.13)	55.87 (11.93)	0.04352
Gender (Female)%	62.35%	85.51%	1.00000
Body Mass Index (BMI)	28.65 (18.43)	27.07 (5.72)	0.49349
Anti-Cyclic Citrullinated Peptide (CCP)	245.25 (470.97)	228.49 (306.47)	0.79905
Clinical Disease Activity Index (CDAI)	5.25 (5.89)	14.53 (12.1)	0.00000
C-Reactive Protein (CRP) (mg/L)	0.34 (0.48)	0.36 (0.48)	0.78621
Health Assessment Questionnaire (HAQ) Score	0.54 (0.58)	1.35 (0.73)	0.00000
Non-Steroidal Anti-Inflammatory Drug (NSAID) Usage%	24.71%	31.88%	1.00000
Rheumatoid Factor (RF)	59.5 (85.95)	146.71 (264.27)	0.00478
Simple Disease Activity Index (SDAI)	6.37 (6.61)	15.6 (11.92)	0.00000
Swollen Joint Count-28 (SJC28)	1.55 (3.47)	3.28 (4.67)	0.00948
Tender Joint Count-28 (TJC28)	1.24 (2.3)	6.01 (6.24)	0.00000
Visual Analog Scale (VAS) Activity (Patient)	20.18 (17.86)	47.52 (25.52)	0.00000
Visual Analog Scale (VAS) Activity (Physician)	12.48 (14.99)	27.73 (21.9)	0.00000
Visual Analog Scale (VAS) Pain	18.38 (17.07)	39.55 (24.49)	0.00000
Erythrocyte Sedimentation Rate (ESR) (mm)	12.8 (10.85)	22.67 (20.37)	0.00018
Disease Activity Score in 28 Joints (DAS28-CRP)	2.35 (0.78)	3.69 (1.28)	0.00000
Disease Activity Score in 28 Joints (DAS28-ESR)	2.34 (0.99)	4.06 (1.36)	0.00000
Asthma %	0.0%	0.0%	1.00000
Inflammatory Bowel Disease %	0.0%	0.0%	1.00000
Prednisolone%	29.41%	49.28%	1.00000
Chronic Renal Insufficiency %	0.0%	0.0%	1.00000
Coronary Heart Disease %	0.0%	0.0%	1.00000
Diabetes %	0.0%	0.0%	1.00000
Fat Metabolism Disorder %	0.0%	0.0%	1.00000
Gout %	0.0%	0.0%	1.00000
Conventional Synthetic Disease-Modifying Antirheumatic Drugs (csDMARD) %	63.53%	71.01%	1.00000

Mean (±standard deviation) and percentage of the population for each variable at baseline for RA patients treated with bDMARDs. ***p***-values indicate the statistical significance of differences between sustained responders and non-sustained responders.

**Table A4:** Configuration space and the best hyper-parameters of each classifier.

Classifier	Hyper-Parameter	Search Space	Response	Sustained
AdaBoost	n_estimators	[5, 10, 50, 100, 200]	5	5
	learning_rate	[0.01, 0.1, 1]	0.01	0.01
SVM	C	[0.1, 1, 10, 100, 1000]	100	100
	gamma	[$1\times 10^{-6}$, $1\times 10^{-5}$, $1\times 10^{-4}$, $1\times 10^{-3}$, $1\times 10^{-2}$, 0.1, 1]	$1\times 10^{-3}$	$1\times 10^{-3}$
KNN	n_neighbors	[1, 3, 5, 7, 8, 10, 12]	3, 6, 7, 8	3, 6, 7, 8
	leaf_size	[1, 50]	1	1
XGBoost	n_estimators	[1, 10, 100, 200]	10, 50, 100, 200	1, 10, 100, 100
	min_child_weight	[1, 5, 10]	1, 5	1, 5
Random Forest	n_estimators	[1, 10, 100]	10, 100	10, 100
	max_features	[‘sqrt’, ‘log2’]	‘sqrt’	‘sqrt’
	max_depth	[1, 2, 3, 4]	1, 3, 4	1, 3, 4

## References

[1] Firestein G.S. (2003). Evolving concepts of rheumatoid arthritis. Nature.

[2] McInnes I.B., Schett G. (2011). The pathogenesis of rheumatoid arthritis. N. Engl. J. Med..

[3] Alamanos Y., Drosos A.A. (2005). Epidemiology of adult rheumatoid arthritis. Autoimmun. Rev..

[4] Smolen J.S., Landewé R.B.M., Bijlsma J.W.J., Burmester G.R., Dougados M., Kerschbaumer A., McInnes I.B., Sepriano A., Van Vollenhoven R.F., De Wit M. (2020). EULAR recommendations for the management of rheumatoid arthritis with synthetic and biological disease-modifying antirheumatic drugs: 2019 update. Ann. Rheum. Dis..

[5] Smolen J.S., Breedveld F.C., Burmester G.R., Bykerk V., Dougados M., Emery P., Kvien T.K., Navarro-Compán M.V., Oliver S., Schoels M. (2016). Treating rheumatoid arthritis to target: 2014 update of the recommendations of an international task force. Ann. Rheum. Dis..

[6] Smolen J.S., Landewé R.B.M., Bergstra S.A., Kerschbaumer A., Sepriano A., Aletaha D., Caporali R., Edwards C.J., Hyrich K.L., Pope J.E. (2023). EULAR recommendations for the management of rheumatoid arthritis with synthetic and biological disease-modifying antirheumatic drugs: 2022 update. Ann. Rheum. Dis..

[7] Pierreisnard A., Issa N., Barnetche T., Richez C., Schaeverbeke T. (2013). Meta-analysis of clinical and radiological efficacy of biologics in rheumatoid arthritis patients naive or inadequately responsive to methotrexate. Jt. Bone Spine.

[8] Combe B., Rincheval N., Benessiano J., Berenbaum F., Cantagrel A., Daurès J.P., Dougados M., Fardellone P., Fautrel B., Flipo R.M. (2013). Five-year favorable outcome of patients with early rheumatoid arthritis in the 2000s: Data from the ESPOIR cohort. J. Rheumatol..

[9] Kievit W., Adang E.M., Fransen J., Kuper H.H., Van De Laar M.A.F.J., Jansen T.L., De Gendt C.M.A., De Rooij D.J.R.A.M., Brus H.L.M., Van Oijen P.C.M. (2008). The effectiveness and medication costs of three anti-tumour necrosis factor *α* agents in the treatment of rheumatoid arthritis from prospective clinical practice data. Ann. Rheum. Dis..

[10] van der Heide A., Jacobs J.W.G., Bijlsma J.W.J., Heurkens A.H.M., van Booma-Frankfort C., van der Veen M.J., Haanen H.C.M., Hofman D.M. (1996). The effectiveness of early treatment with “second-line” antirheumatic drugs: A randomized, controlled trial. Ann. Intern. Med..

[11] Aletaha D., Kapral T., Smolen J.S. (2003). Toxicity profiles of traditional disease modifying antirheumatic drugs for rheumatoid arthritis. Ann. Rheum. Dis..

[12] Antoni C., Braun J. (2002). Side effects of anti-TNF therapy: Current knowledge. Clin. Exp. Rheumatol..

[13] De La Torre I., Valor L., Nieto J.C., Hernandez D., Martinez L., Gonzalez C.M., Monteagudo I., Longo J.L., Montoro M., Carreño L. (2013). Anti-TNF treatments in rheumatoid arthritis: Economic impact of dosage modification. Expert Rev. Pharmacoecon. Outcomes Res..

[14] Kim Y., Kim G.T., Suh Y.S., Kim H.O., Lee H.N., Lee S.G. (2020). The impact of the amendment of the Korean national health insurance reimbursement criteria for anti-tumor necrosis factor-*α* agents on treatment pattern, clinical response and persistence in patients with rheumatoid arthritis. J. Rheum. Dis..

[15] Batticciotto A., Ravasio R., Riva M., Sarzi-Puttini P. (2016). Efficacy and treatment costs of monotherapy with bDMARDs in the treatment of rheumatoid arthritis in patients intolerant to or inappropriate to continue treatment with methotrexate. Adv. Ther..

[16] Jordan M.I., Mitchell T.M. (2015). Machine learning: Trends, perspectives, and prospects. Science.

[17] Wei M., Chu C.-Q. (2022). Prediction of Treatment Response: Personalized Medicine in the Management of Rheumatoid Arthritis. Best Pract. Res. Clin. Rheumatol..

[18] Koo B.S., Eun S., Shin K., Yoon H., Hong C., Kim D.H., Hong S., Kim Y.G., Lee C.K., Yoo B. (2021). Machine learning model for identifying important clinical features for predicting remission in patients with rheumatoid arthritis treated with biologics. Arthritis Res. Ther..

[19] Lee S., Kang S., Eun Y., Won H.H., Kim H., Lee J., Koh E.M., Cha H.S. (2021). Machine learning-based prediction model for responses of bDMARDs in patients with rheumatoid arthritis and ankylosing spondylitis. Arthritis Res. Ther..

[20] Guan Y., Zhang H., Quang D., Wang Z., Parker S.C.J., Pappas D.A., Kremer J.M., Zhu F. (2019). Machine learning to predict anti-tumor necrosis factor drug responses of rheumatoid arthritis patients by integrating clinical and genetic markers. Arthritis Rheumatol..

[21] Tao W., Concepcion A.N., Vianen M., Marijnissen A.C.A., Lafeber F.P.G.J., Radstake T.R.D.J., Pandit A. (2021). Multiomics and machine learning accurately predict clinical response to adalimumab and etanercept therapy in patients with rheumatoid arthritis. Arthritis Rheumatol..

[22] Rivellese F., Surace A.E.A., Goldmann K., Sciacca E., Çubuk C., Giorli G., John C.R., Nerviani A., Fossati-Jimack L., Thorborn G. (2022). Rituximab versus Tocilizumab in Rheumatoid Arthritis: Synovial Biopsy-Based Biomarker Analysis of the Phase 4 R4RA Randomized Trial. Nat. Med..

[23] Yoosuf N., Maciejewski M., Ziemek D., Jelinsky S.A., Folkersen L., Müller M., Sahlström P., Vivar N., Catrina A., Berg L. (2022). Early Prediction of Clinical Response to Anti-TNF Treatment Using Multi-Omics and Machine Learning in Rheumatoid Arthritis. Rheumatology.

[24] Kay J., Upchurch K.S. (2012). ACR/EULAR 2010 rheumatoid arthritis classification criteria. Rheumatology.

[25] England B.R., Sokolove J., Robinson W.H., Thiele G.M., Ganti A.K., Sayles H., Michaud K., Caplan L., Davis L.A., Cannon G.W. (2016). Associations of circulating cytokines and chemokines with cancer mortality in men with rheumatoid arthritis. Arthritis Rheumatol..

[26] Mongin D., Lauper K., Turesson C., Hetland M.L., Kristianslund E.K., Kvien T.K., Santos M.J., Pavelka K., Iannone F., Finckh A. (2019). Imputing Missing Data of Function and Disease Activity in Rheumatoid Arthritis Registers: What is the Best Technique?. RMD Open.

[27] Jakobsen J.C., Gluud C., Wetterslev J., Winkel P. (2017). When and How Should Multiple Imputation Be Used for Handling Missing Data in Randomised Clinical Trials: A Practical Guide with Flowcharts. BMC Med. Res. Methodol..

[28] van Buuren S. (2018). Flexible Imputation of Missing Data.

[29] Hayati Rezvan P., Lee K.J., Simpson J.A. (2015). The Rise of Multiple Imputation: A Review of the Reporting and Implementation of the Method in Medical Research. BMC Med. Res. Methodol..

[30] Felson D.T., Smolen J.S., Wells G., Zhang B., Van Tuyl L.H.D., Funovits J., Aletaha D., Allaart C.F., Bathon J., Bombardieri S. (2011). American College of Rheumatology/European League Against Rheumatism provisional definition of remission in rheumatoid arthritis for clinical trials. Arthritis Rheumatol..

[31] Studenic P., Aletaha D., de Wit M., Stamm T.A., Alasti F., Lacaille D., Smolen J.S., Felson D.T. (2023). American College of Rheumatology/EULAR remission criteria for rheumatoid arthritis: 2022 revision. Ann. Rheum. Dis..

[32] Durgesh K.S., Lekha B. (2010). Data classification using support vector machine. J. Theor. Appl. Inf. Technol..

[33] Liaw A., Wiener M. (2015). randomForest: Breiman and Cutler’s random forests for classification and regression. R Package Version.

[34] Zhang L., Zhan C. (2017). Machine learning in rock facies classification: An application of XGBoost. Proceedings of the International Geophysical Conference.

[35] Lodha S.K., Fitzpatrick D.M., Helmbold D.P. (2007). Aerial lidar data classification using adaboost. Proceedings of the Sixth International Conference on 3-D Digital Imaging and Modeling (3DIM 2007).

[36] Peterson L.E. (2009). K-nearest neighbor. Scholarpedia.

[37] Chen R.-C., Dewi C., Huang S.-W., Caraka R.E. (2020). Selecting critical features for data classification based on machine learning methods. J. Big Data.

[38] Lundberg S.M., Lee S.-I. (2017). A unified approach to interpreting model predictions. Adv. Neural Inf. Process. Syst..

[39] Shapley L.S. (1953). A Value for N-Person Games.

[40] Cawley G.C., Talbot N.L.C. (2010). On over-fitting in model selection and subsequent selection bias in performance evaluation. J. Mach. Learn. Res..

[41] Scheda R., Diciotti S. (2022). Explanations of Machine Learning Models in Repeated Nested Cross-Validation: An Application in Age Prediction Using Brain Complexity Features. Appl. Sci..

[42] Zhong Y., He J., Chalise P. (2020). Nested and repeated cross validation for classification model with high-dimensional data. Rev. Colomb. Estad..

